# Improved survival of patients with newly diagnosed oligometastatic prostate cancer through intensified multimodal treatment

**DOI:** 10.3389/fonc.2024.1475914

**Published:** 2024-12-10

**Authors:** Viktoria Schütz, Christopher-Leo Nessler, Anette Duensing, Stefanie Zschäbitz, Dirk Jäger, Jürgen Debus, Markus Hohenfellner, Stefan Duensing

**Affiliations:** ^1^ Department of Urology, Heidelberg University Hospital, Heidelberg, Germany; ^2^ Precision Oncology of Urological Malignancies, Department of Urology, Heidelberg University Hospital, Heidelberg, Germany; ^3^ Department of Medical Oncology, National Center for Tumor Diseases Heidelberg, Heidelberg University Hospital, Heidelberg, Germany; ^4^ Department of Radiation Oncology, Heidelberg University Hospital, Heidelberg, Germany; ^5^ Molecular Urooncology, Department of Urology, Heidelberg University Hospital, Heidelberg, Germany

**Keywords:** oligometastatic prostate cancer, radical prostatectomy, multimodal treatment, intensified treatment, prostate cancer, hormone sensitive prostate cancer

## Abstract

**Background and objectives:**

The standard of care for patients with metastatic hormone-sensitive prostate cancer (mHSPC) includes androgen deprivation therapy (ADT), novel antihormonal therapies (NHT) and/or chemotherapy. Patients with newly diagnosed oligometastatic prostate cancer (omPCa) represent a distinct subgroup of mHSPC, for which the optimal treatment, particularly the role of radical prostatectomy (RP) and metastasis-directed therapy (MDT), is currently under debate.

**Materials and methods:**

In this single center, retrospective analysis, 43 patients with newly diagnosed omPCa were included. All patients underwent RP as part of a multimodal, personalized treatment approach. Other treatments included ADT, NHT, MDT (surgery or radiotherapy), adjuvant radiotherapy (prostatic fossa and/or pelvic lymph nodes) or chemotherapy in various combinations. Clinical endpoints were progression free and cancer specific survival (PFS, CSS).

**Results:**

No patient with omPCa died from prostate cancer during an up to ten years follow-up period after intensified multimodal treatment i.e., RP, ADT, adjuvant radiation therapy and MDT (n=13). In contrast, patients requiring chemotherapy (n=10) showed a significantly worse PFS (p<0.001) and CSS (p<0.001). Patients receiving various combinations (<4 therapeutic modalities; n=20) showed a more favorable outcome than patients receiving chemotherapy, but differences in PFS and CSS were not statistically significant compared to patients receiving an intensified multimodal treatment.

**Conclusions:**

An intensified, multimodal treatment approach including RP can lead to excellent survival outcomes in patients with newly diagnosed omPCa. Patients requiring chemotherapy have most likely a more aggressive disease and therefore a more rapid tumor progression. Future studies to identify markers for risk stratification in patients with omPCa are therefore needed.

## 1 Introduction

Prostate cancer (PCa) is the most common non-cutaneous malignancy in Western men (1). While cure rates are excellent for patients with localized disease, patients presenting with synchronous or metachronous metastasis have a poor prognosis and typically only palliative treatment options (2). However, there is a subgroup of patients with synchronous metastatic dissemination limited to a maximum of four bone lesions i.e., oligometastatic prostate cancer (omPCa), in which a curative therapeutic approach appears to be a viable option (3).

For localized PCa, radical prostatectomy (RP) or radiation therapy (RT) are the standard treatment modalities according to current guidelines (4). Treatment options for metastatic hormone sensitive prostate cancer (mHSPC) have expanded significantly in recent years and now include classical androgen deprivation therapy (ADT), novel antihormonal agents (NHT) and taxane-based chemotherapy (Cx). It has recently been shown that the triple therapy of ADT, NHT and Cx leads to a better survival outcome than ADT in combination with docetaxel (5). These results underscore that an early intensified treatment can lead to a substantial survival advantage.

For patients with newly diagnosed, synchronous omPCa treatment options are currently under debate (3). The concept of oligometastatic disease was first introduced by Helman and Weichselbaum for patients with a limited number of metastases (6) in which local treatment and metastasis-directed therapy (MDT) can improve survival. To our knowledge, there is no consensus definition of newly diagnosed omPCa in the literature (7, 8) or current guidelines (4, 9). Another aspect to consider is not only the lack of a clear definition of omPCa, making the data available inconsistent, but also the change in imaging modalities over the past years with an increased use of PSMA-PET-CT (10). With PSMA-PET-CT being more sensitive than conventional imaging, metastases can be detected earlier in the course of the disease (11), which could lead to an increased incidence of omPCa (3), and hence the number of patients for whom an adequate treatment needs to be established.

In the past several years, local treatment for omPCa including cytoreductive RP and RT have been discussed extensively (12–14). Local RT in combination with systemic treatment has been shown to improve overall survival (OS) as well as progression free survival (PFS) in comparison to systemic treatment alone in patients with a low metastatic burden (15–17) and has since been established as a standard treatment. However, studies on RP in patients with omPCa are limited (18). Those limited studies show trends towards improvement in overall, progression free and cancer specific survival (CSS) (12, 19–21).

In addition to systemic treatment, RT and RP, the concept of MDT is emerging (22, 23) and has recently been reviewed by Miszcyk et al. (24). Current guidelines do not include MDT in metastatic PCa patients (4, 9). However, there are data to suggest that MDT extends the time until systemic treatment is required (25), might improve oncological outcome (26–29) and can lead to local symptoms control (24). MDT commonly includes surgical resection and RT (24), however, randomized, prospective clinical trials on the role of MDT in omPCa are largely lacking.

With omPCa being discussed as an intermediate state between localized and a disseminated disease (6), a multimodal treatment approach including RP or RT, MDT and systemic treatment appears to be particularly promising (3). In this retrospective, single-center analysis, we investigate the long-term oncological outcome of patients who underwent multimodal treatment for newly diagnosed omPCa combining RP, MDT as well as systemic treatment, as part of a highly personalized therapeutic approach.

## 2 Materials and methods

### 2.1 Patient population

Forty-three patients with newly diagnosed omPCa were included in this retrospective, single-center analysis. Patients had a maximum of four bone metastases and/or non-regional lymph node metastases (up to a maximum of two) (30). Patients with visceral metastasis (cM1c) were excluded. All patients underwent RP between 2000 and 2022 as part of an individual, personalized treatment strategy. Other treatment modalities included ADT, NHT, surgery or RT of metastases, adjuvant RT of the prostatic fossa and/or pelvic lymphatics and taxane-based Cx in various combinations. Endpoints were PFS (defined as biochemical recurrence, PSA progression, radiographic progression of metastases, development of new metastases or local recurrence) and CSS. Maximum follow-up time was 140 months with a median follow-up of 69 months (range 4-140).

Patient information was retrieved from the clinical information system of the University Hospital Heidelberg and from the tumor database of the Department of Urology, a prospective database collecting clinical, imaging and pathological data of every patient with an urological malignancy. This data base also includes prospectively collected follow-up data. Patients gave written informed consent for the use of their data for research and publication. This analysis was approved by the ethics committee of the Medical Faculty Heidelberg of the University of Heidelberg (S-335/2021).

### 2.2 Statistical analyses

To assess statistical significance the Chi-square test and Kruskal Wallis test were used. A p value of <0.05 was considered significant. The Kaplan-Meier method was used to calculate CSS and PFS with log-rank statistics. Descriptive analysis was done using Microsoft Excel Version 2411 and statistical analysis was completed using IBM SPSS Statistics for Windows, Version 27 and Version 29 (IBM Corp., Armonk, N.Y., USA).

## 3 Results

### 3.1 Baseline patient characteristics

A total of 43 patients with newly diagnosed omPCa were included in this analysis. All patients presented with four or less bone metastases (n=38), non-regional lymph node metastases (n=5) but no visceral metastases. A combination of bone and non-regional lymph node metastases was present in six patients (13.9%). All patients had biopsy proven adenocarcinoma of the prostate with a median initial PSA level of 22.9 ng/ml (range, 4.8-599.6 ng/ml). Basic patient characteristics are shown in **Table 1**.

**Table 1:** Basic patient characteristics (n= 43).

Characteristics	Results
Age at initial diagnosis, years	
Median (Range)	62 (43-76)
BMI	
Median (Range)	27 (21-35)
Initial PSA level, ng/ml	
Median, Range	22.9 (4.8-559.6)
ECOG performance-status, n (%)	
0	40 (93)
1	3 (7)
Grade Group (Gleason Score Biopsy), n (%)	
1 (3 + 3)	3 (7.0)
2 (3 + 4)	4 (9.3)
3 (4 + 3)	3 (7.0)
4 (4 + 4)	10 (23.3)
5 (9 and 10)	22 (51.2)
Missing	1 (2.3)
Staging, n (%)	
MRI, CT, bone scan	19 (44.2)
PSMA-PET-CT	24 (55.8)
pT, n (%)	
2c	5 (12)
3a	8 (18)
3b	27 (63)
4	3 (7)
pN, n (%)	
N0	15 (35)
N+	28 (65)
c/pM, n (%)	
M1a only	5 (12)
M1b	38 (88)
Number of non-regional lymph node metastases in patients stage M1b, n (%)	
0	41 (95.4)
1	1 (2.3)
2	1 (2.3)
Number of bone metastases, n (%)	
0	5 (12)
1	20 (47)
2	8 (19)
3	6 (14)
4	4 (9)
R status, n (%)	
R0	14 (33)
R1	29 (67)
Grade Group (Gleason Score Biopsy), n (%)	
2 (3 + 4)	3 (7.0)
3 (4 + 3)	13 (30.2)
4 (4 + 4)	1 (2.3)
5 (4 + 5)	26 (60.5)
Surgical technique, n (%)	
Open	24 (56)
Robotic	19 (44)

### 3.2 Feasibility of multimodal therapy in patients with omPCa

**Figure 1:**
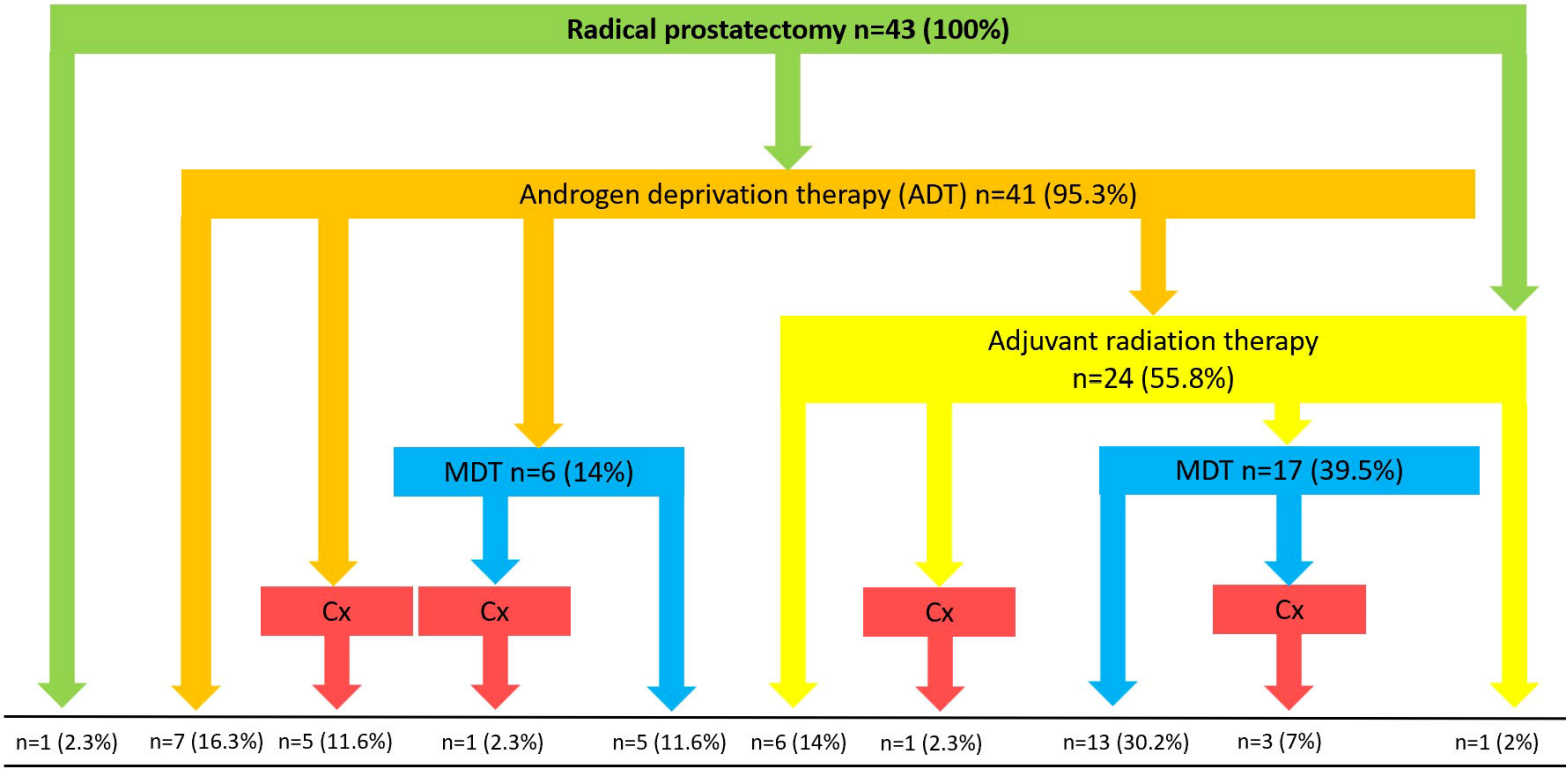
Overview of different treatment combinations. MDT, Metastasis-directed therapy; Cx, Chemotherapy.

All patients (n=43) underwent RP as part of a multimodal, individualized therapeutic approach. A total of 24 patients (55.8%) received adjuvant radiation therapy after surgery. ADT was administered in 41 patients (95.3%). In addition to RP, RT and ADT, a total of 23 patients received MDT (53.5%). A total of ten patients required chemotherapy during the course of disease (23.3%). An overview of the different treatment combinations patients received is shown in **Figure 1**.

**Figure 2:**
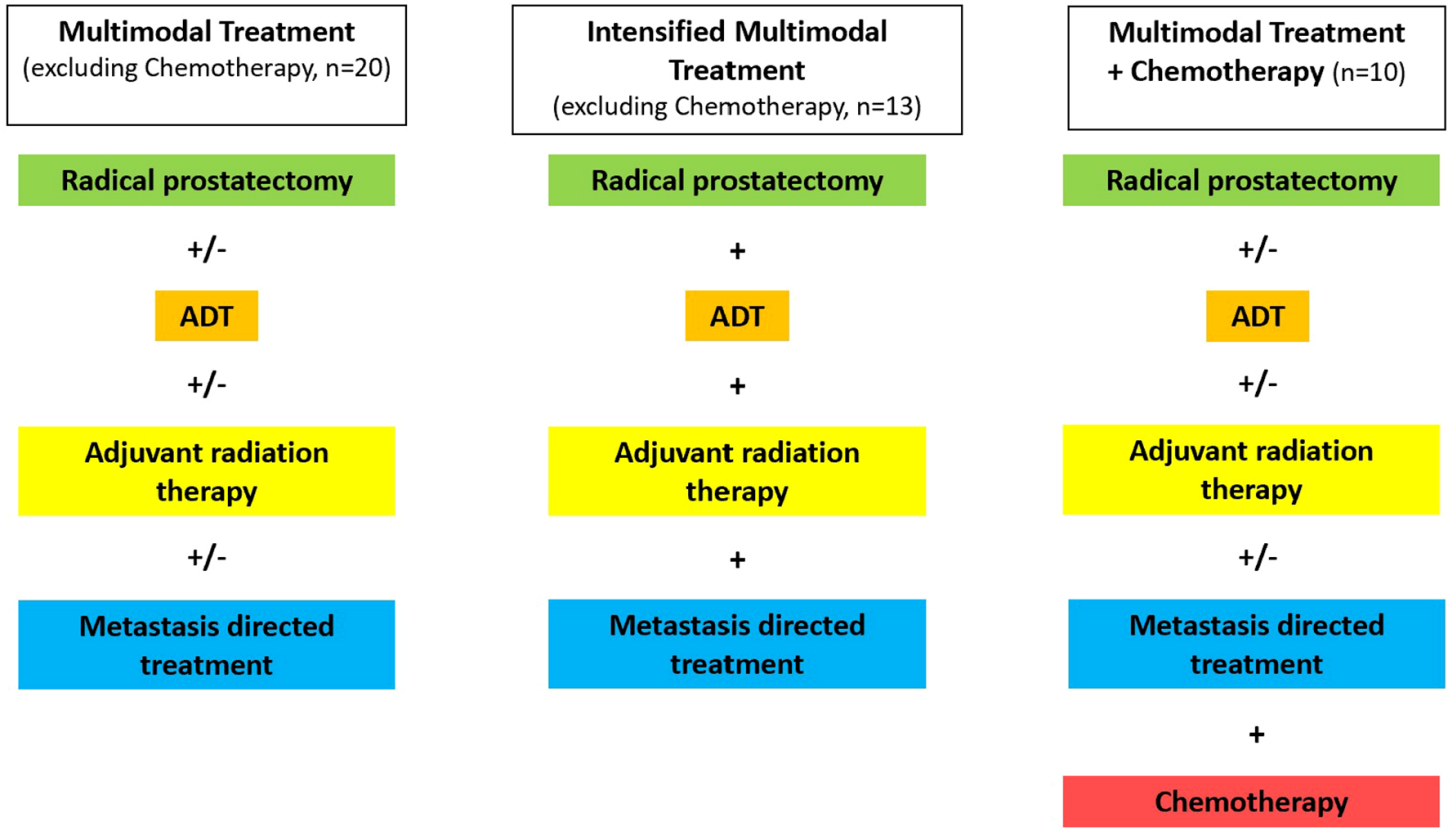
Definition of three different treatment groups, defined by the combination of treatment modalities patients received as part of a multimodal treatment for oligometastatic prostate cancer. ADT, Androgen deprivation therapy; MDT, Metastasis-directed therapy; Cx, Chemotherapy.

Based on the different treatment modalities three different patient groups were identified as shown in **Figure 2**. Patients in group one (n= 20, 46.5%) received one (n=1, 2.3%), two or more of the different treatment modalities in different combinations. Patients in group two (n= 13, 3.2%) received an intensified multimodal treatment including all of various treatment options available excluding chemotherapy. Patients in group three (n=10, 23.3%) received a taxane-based chemotherapy in addition to various combinations of RP, adjuvant RT, ADT and MDT at some point during the course of the disease.

Clinico-pathological characteristics did not significantly differ and were comparable between the three patient subgroups (**Table 2**).

**Table 2:** Clinico-pathologic characteristics of patients with or without intensified, multimodal treatment.

	Multimodal Treatment (RP ± ADT ± RT ± MDT; various combinations excluding Cx)	Intensified Multimodal Treatment (RP + ADT + RT + MDT; excluding Cx)	Multimodal Treatment incl. Chemotherapy	*p-value *
Patients, n	20	13	10	
Age, years				0.340
Median (Range)	62.5 (50-72)	59.0 (43-76)	61.5 (52-66)	
Initial PSA (ng/ml)				0.250
Median, Range	25.5 (8.1-559.6)	15.2 (4.8-191.4)	27.8 (6.6-130.0)	
Staging, n (%)				0.150
MRI, CT, bone scan	8 (40.0)	4 (30.8)	7 (70.0)	
PSMA-PET-CT	12 (60.0)	9 (69.2)	3 (30.0)	
Grade Group (Gleason Score Biopsy), n (%)				0.295
2 and 3 (3+4 and 4+3)	10 (50.0)	3 (23.1)	7 (30.0)	
4 (8)	0	0	1 (10.0)	
5 (9 and 10)	10 (50.0)	10 (76.9)	6 (60.0)	
pT, n (%)				0.130
2c	4 (20.0)	1 (7.7)	0	
3a	5 (25.0)	1 (7.7)	2 (20.0)	
3b	10 (50.0)	11 (84.6)	6 (60.0)	
4	1 (5.0)	0	2 (20.0)	
pN, n (%)				0.406
N0	9 (45.0)	3 (23.1)	3 (30.0)	
N+	11 (55.0)	10 (76.9)	7 (70.0)	
Number of lymph nodes resected				0.247
Median (Range)	25 (9-44)	33 (16-48)	33.5 (7-56)	
c/pM, n (%)				0.424
M1a	2 (20.0)	2 (15.4)	
M1b	19 (95.0)	11 (84.6)	8 (80.0)	
Bone metastases, n (%)				0.318
0	1 (5.0)	2 (15.4)	2 (20.0)	
1	9 (45.0)	7 (53.8)	4 (40.0)	
2	4 (20.0)	3 (23.1)	1 (10.0)	
3	3 (15.0)	1 (7.7)	2 (20.0)	
4	3 (15.0)	0	1 (10.0)	
R status, n (%)				0.177
0	7 (35.0)	6 (46.2)	1 (10.0)	
1	13 (65.0)	7 (53.8)	9 (90.0)	
Surgical technique, n (%)				0.280
Open	12 (60.0)	5 (38.5)	7 (70.0)	
Robotic	8 (40.0)	8 (61.5)	3 (30.0)	

RP, radical prostatectomy; ADT, androgen deprivation therapy; RT, radiation therapy; MDT, metastasis-directed therapy; Cx, Chemotherapy.

### 3.3 Survival advantage in patients receiving an intensified multimodal treatment

To ascertain the impact of an intensified multimodal treatment on the oncological outcome, PFS and CSS were compared between the three patient subgroups.

Of the 43 patients, 25 patients experienced disease progression. 21 patients (48.8%) had a biochemical recurrence (BCR), with or without a local recurrence and/or new metastases. Three patients (7%) experienced PSA progression and one patient (2.3%) died from progressive prostate cancer.

**Figure 3:**
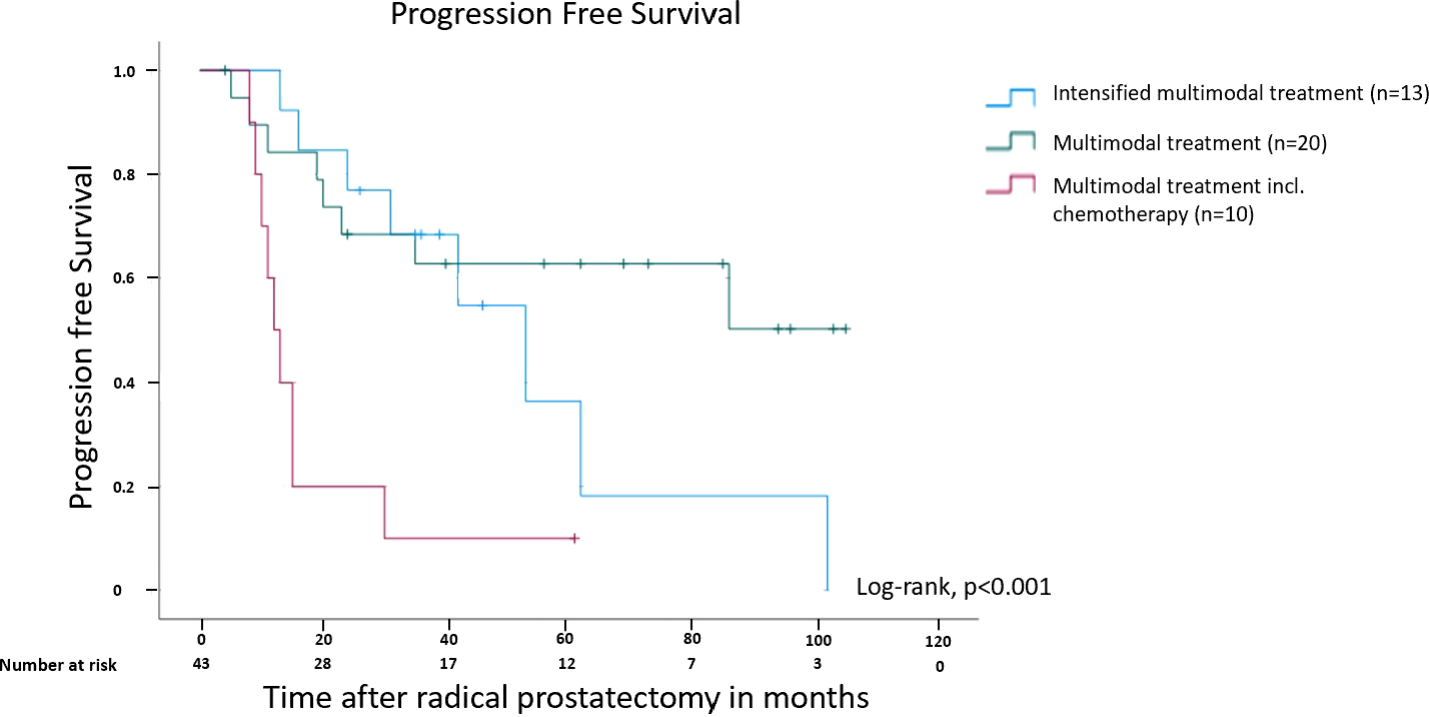
Kaplan-Meier curve showing progression free survival in months after radical prostatectomy for patients receiving multimodal treatment, multimodal treatment including chemotherapy and intensified multimodal treatment. Please see a definition of subgroups in **Figure 2**.

Patients receiving an intensified multimodal therapy (all treatment modalities available except Cx) showed a more favorable PFS compared to patients receiving multimodal therapy (less than the maximum number of treatment modalities, except Cx). However, it needs to be emphasized that the differences in PFS between the intensified multimodal and the multimodal therapy group were not statistically significant (p=0.277). The Kaplan-Meier curve for PFS of all three patient subgroups clearly shows the survival disadvantage of patients requiring chemotherapy in comparison to the other two subgroups (p<0.001; **Figure 3**). The 5-year PFS rates in the intensified multimodal treatment group, multimodal treatment group and chemotherapy group were 53.8%, 65% and 10%, respectively.

**Figure 4:**
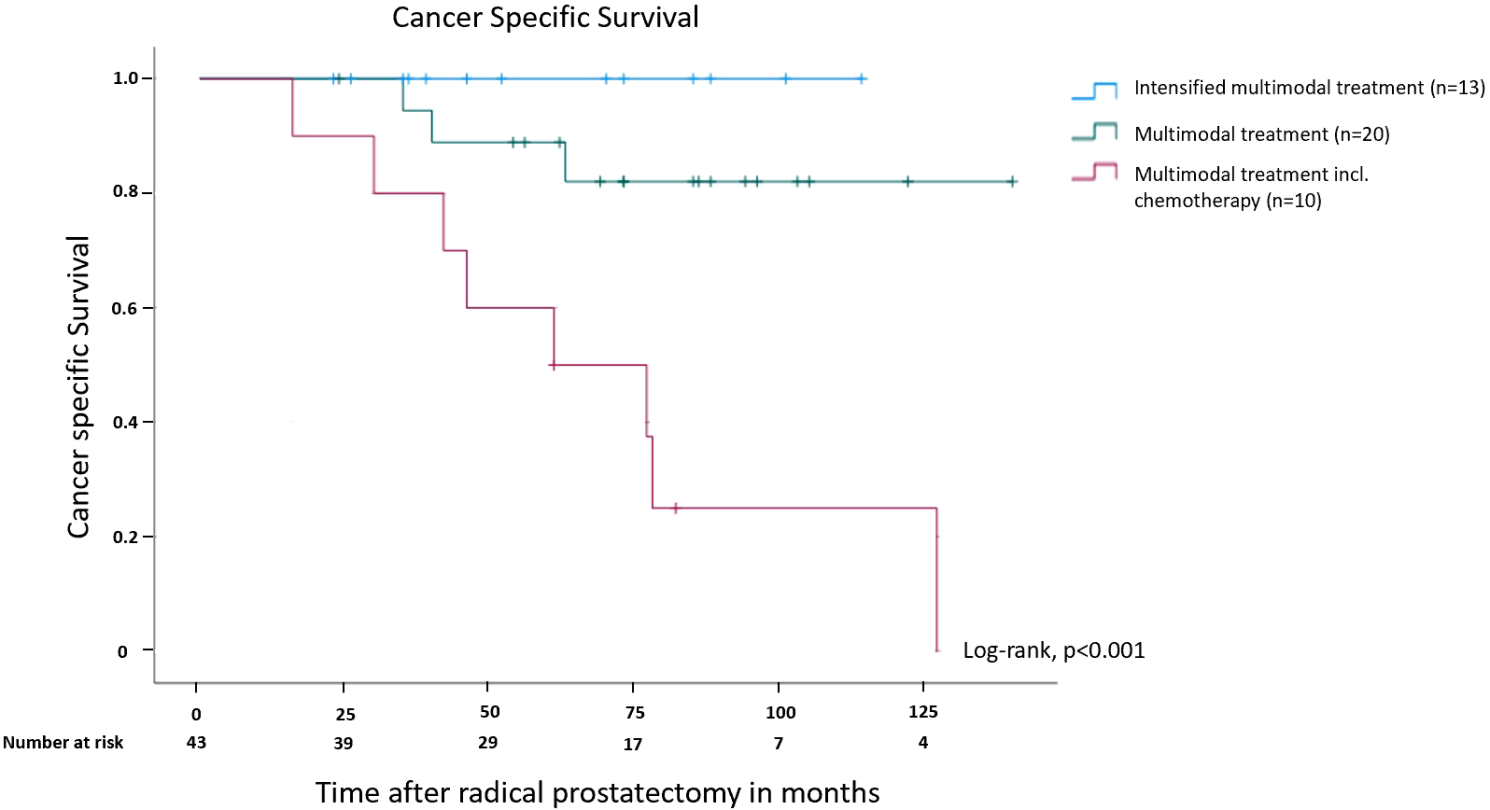
Kaplan-Meier curve showing cancer specific survival in months after radical prostatectomy for patients receiving multimodal treatment, multimodal treatment including chemotherapy and intensified multimodal treatment. Please see a definition of subgroups in **Figure 2**.

Patients receiving an intensified multimodal treatment showed a significantly better CSS compared to the other two patient groups (**Figure 4**). None of the patients in the intensified multimodal treatment group (n=13) died from prostate cancer during a follow-up period of up to 140 months. The Kaplan-Meier curve for CSS of all three patient subgroups demonstrates the survival disadvantage of patients requiring chemotherapy in comparison to the other two subgroups (p<0.001), while differences in CSS between the intensified multimodal treatment group and the multimodal treatment group were not statistically significant (p=0.215; **Figure 4**). The 5- and 10-year CSS rate was 100% in the intensified multimodal treatment group. In patients receiving a less intensified treatment, the 5- and 10-year CSS rate was 90% and 85%, respectively. In the chemotherapy group, the 5- and 10-year CSS rate was 60% and 20%, respectively.

A multivariate Cox regression analysis for PFS and CSS showed that only the Biopsy Grade Group (<4 vs. ≥4; p=0.007) and the number of bone metastases (<3 vs. ≥3; p=0.021) were independent prognosticators for the PFS (**Supplementary Table 1**).

Taken together, these results show that patients with omPCa benefit significantly from an intensified multimodal treatment including RP, RT, ADT and MDT.

## 4 Discussion

Oligometastatic PCa represents a rare subgroup of metastatic PCa. Treatment strategies for this distinct patient subgroup are under intensive debate. In particular the role of RP remains unclear (31–35) while RT has been accepted as a local treatment option for patients with omPCa as a result of the STAMPEDE trial (36). Importantly, with PSMA-PET-CT being more widely used for PCa staging (11), the number of patients with newly diagnosed omPCa is very likely to rise.

In this retrospective analysis, we evaluated the oncological outcome of 43 patients with *de novo* omPCa. All patients underwent RP between 2000 and 2022 in addition to a combination of further treatment modalities including RT, ADT, MDT and Cx in an individualized treatment approach. Three different patient and treatment groups were defined depending on the treatments received in addition to RP. Patients receiving an intensified multimodal treatment i.e., a combination of RP, RT, ADT, and MDT (n=13) showed a superior survival with 5- and 10-year CSS rates of 100%. In contrast, the outcome for patients requiring chemotherapy during the course of the disease was significantly worse suggesting a more aggressive biology.

To our knowledge, there is no consensus definition of omPCa based on the literature or current guidelines (4, 9). In our study, we decided to choose criteria suggested by the CHAARTED trial i.e., a maximum of four bone metastatic sites (7), however, with inclusion of non-regional lymph-node metastasis (M1a). The rationale for the latter is based on the evidence that patients with stage M1a have a worse prognosis than patients with stage N1 (37). Less than five bone metastases have been used by a number of other studies such as the STAMPEDE (15) or HORRAD (16) trial.

In contrast to RT, RP is not recommended by current guidelines for patients with omPCa (4, 9). The feasibility and safety of RP in patients with metastatic PCa has been previously shown (21, 33, 34) including its role for cytoreduction (12, 13, 38) and local symptom control in a palliative setting (13).

In a study comparing the oncological outcome of 78 PCa patients with a low metastatic burden undergoing RP, similar results (CSS rate of 92% after three years) compared to patients from the STAMPEDE trial (Arm H; CSS rate of 86% after three years) could be achieved (39). An evaluation of data from the SEER database showed that men with metastatic PCa undergoing RP (n=47) or RT (n=88) of the prostate show a decrease in PCa specific mortality (RP=52% and RT=62% risk reduction in cancer specific mortality) compared to patients receiving ADT only (40).

In our study, more than 50% of the patients received an adjuvant RT because of negative predictive factors. This raises the question whether a primary RT would be an option in this patient population. In addition to what already has been discussed we would like to point out that a recent evaluation of RT and RP in the treatment of omPCa patients did not show a significant difference in regard to 5-year PFS and OS between the two treatment modalities (41). An important aspect to consider is the risk of local recurrence – especially in patients with locally advanced disease. Hence, these patients would require a salvage RP after RT, which has been shown to be associated with a higher risk for complications when compared to primary RP (42). We therefore believe that our approach to perform RP in patients with omPCa is not only feasible and leads to favorable clinical results while avoiding the adverse events associated with salvage RP.

Besides RP, the role of MDT has also not yet been established in the management of omPCa patients (4). In our analysis, 23 patients received MDT, which in combination with RP, RP and ADT resulted in an excellent CSS over a time period of ten years. MDT has been discussed in the treatment of PCa patients with a low metastatic burden as part of a multimodal treatment strategy (23, 26). In PCa patients with recurrent disease and nodal involvement, local resection or RT has been shown to lead to an improvement in CSS compared to ADT alone (27). Our results are further supported by studies showing an improvement in ADT-free survival and PFS comparing MDT vs. surveillance in recurrent PCa (26, 28). In another analysis of 68 patients with omPCa, patients additionally receiving MDT (n=24; surgical resection or radiation therapy within six months after RP) showed a significantly reduced mortality rate (p = 0.04) compared to patients who did not receive MDT (29). These results as well as results from our analysis emphasize the need for a further evaluation of MDT in omPCa patients. A study that might further support the use of MDT is the ongoing PERSIAN trial (NCT05717660), a prospective, multicentric Phase II randomized superiority study evaluating the role of radiation therapy on all metastatic sites in combination with ADT and apalutamide compared to ADT and apalutamide alone (43).

With PSMA-PET-CT being used more widely, omPCa is likely to be detected more frequently. This underscores the need for consensus treatment strategies for these patients. Already in 2016 it has been discussed that some patients with early stage metastatic PCa might benefit from a multimodal treatment strategy (44), which is supported by the results of the present analysis. However, our study suggests that a subset of patients, in particular patients requiring chemotherapy for rapid disease progression, benefit less or do not benefit from an intensified multimodal treatment. It will be important in future trials to develop suitable biomarkers to identify these patients, for example genetic testing for pathogenic mutations in *BRCA1/2* or *TP53* (45).

Limitations of our analysis include the single-center, retrospective character and the relatively small patient cohort. Another limitation is the heterogeneity of pre-operative imaging modalities. It is well established that PSMA-PET-Scans show a higher sensitivity and specificity than conventional imaging (11). Hence, patients staged by conventional imaging may experience an underestimate of the true metastatic burden. The importance of a more accurate clinical staging has recently been demonstrated, with patients receiving a PSMA-PET-CT before radiation therapy showing a significantly better 5-year CSS (46). However, there were no statistically significant differences between the three patient subgroups of our study with respect to imaging modalities (p=0.150; **Table 2**). Moreover, there was no difference in patient PFS (p=0.143) and CSS (p=0.078) depending on the imaging modalities used.

The change of the therapeutic landscape for patients with metastatic prostate cancer in the past decades also needs to be taken into consideration when interpreting our results. At the same time, future studies should also consider novel systemic therapy as part of multimodal treatment approach for omPCa e.g., PARP inhibitors (47).

Taking into account the results of this investigation and data available in current literature, it seems feasible and promising to consider an intensified multimodal treatment approach including RP for patients with newly diagnosed omPCa which can lead to a long-term survival benefit. The challenge is also going to be to correctly identify patients that are going to benefit from these treatment strategies, as well as those in need of a more aggressive, systemic treatment regimen.

## 5 Conclusion

Intensified multimodal treatment for newly-diagnosed omPCa leads to excellent survival results. However, patients requiring chemotherapy do not seem to benefit, possibly due to a more aggressive disease. Further studies are needed to help identify patients benefitting from intensified, multimodal treatment.

## Data Availability

The original contributions presented in the study are included in the article/**Supplementary Material**. Further inquiries can be directed to the corresponding author.

## References

[1] Siegel RL Miller KD Wagle NS Jemal A. Cancer statistics, 2023. CA: A Cancer J Clin. (2023) 73:17–48. doi: 10.3322/caac.21763 36633525

[2] Ong S O'Brien J Medhurst E Lawrentschuk N Murphy D Azad A. Current treatment options for newly diagnosed metastatic hormone-sensitive prostate cancer-a narrative review. Transl Androl Urol. (2021) 10:3918–30. doi: 10.21037/tau-20-1118 PMC8575582 34804835

[3] Juan GR Laura FH Javier PV Natalia VC Isabel MGR Enrique RG. Where do we stand in the management of oligometastatic prostate cancer? A comprehensive review. Cancers (Basel). (2022) 14. doi: 10.3390/cancers14082017 PMC9029666 35454924

[4] Mottet N Cornford P van den Bergh RCN Briers E Eberli D De Meerleer G. EAU - EANM - ESTRO - ESUR - ISUP - SIOG Guidelines on Prostate Cancer. Arnhem, The Netherlands: EAU Guidelines Office (2023).

[5] Smith MR Hussain M Saad F Fizazi K Sternberg CN Crawford ED. Darolutamide and survival in metastatic, hormone-sensitive prostate cancer. New Engl J Med. (2022) 386:1132–42. doi: 10.1056/NEJMoa2119115 PMC9844551 35179323

[6] Hellman S Weichselbaum RR. Oligometastases. J Clin Oncol. (1995) 13:8–10. doi: 10.1200/JCO.1995.13.1.8 7799047

[7] Sweeney CJ Chen YH Carducci M Liu G Jarrard DF Eisenberger M. Chemohormonal therapy in metastatic hormone-sensitive prostate cancer. New Engl J Med. (2015) 373:737–46. doi: 10.1056/NEJMoa1503747 PMC4562797 26244877

[8] Fizazi K Tran N Fein L Matsubara N Rodriguez-Antolin A Alekseev BY. Abiraterone plus prednisone in metastatic, castration-sensitive prostate cancer. N Engl J Med. (2017) 377:352–60. doi: 10.1056/NEJMoa1704174 28578607

[9] Lowrance W Dreicer R Jarrard DF Scarpato KR Kim SK Kirkby E. Updates to advanced prostate cancer: AUA/SUO guideline (2023). J Urol. (2023) 209:1082–90. doi: 10.1097/JU.0000000000003452 37096583

[10] Rogowski P Roach M 3rd Schmidt-Hegemann NS Trapp C von Bestenbostel R Shi R. Radiotherapy of oligometastatic prostate cancer: a systematic review. Radiat Oncol. (2021) 16:50. doi: 10.1186/s13014-021-01776-8 33750437 PMC7941976

[11] Hofman MS Lawrentschuk N Francis RJ Tang C Vela I Thomas P. Prostate-specific membrane antigen PET-CT in patients with high-risk prostate cancer before curative-intent surgery or radiotherapy (proPSMA): a prospective, randomised, multicentre study. Lancet. (2020) 395:1208–16. doi: 10.1016/S0140-6736(20)30314-7 32209449

[12] Heidenreich A Pfister D. Radical cytoreductive prostatectomy in men with prostate cancer and oligometastatic disease. Curr Opin Urol. (2020) 30:90–7. doi: 10.1097/MOU.0000000000000691 31724996

[13] Lumen N De Bleser E Buelens S Verla W Poelaert F Claeys W. The role of cytoreductive radical prostatectomy in the treatment of newly diagnosed low-volume metastatic prostate cancer. Results from the local treatment of metastatic prostate cancer (LoMP) registry. Eur Urol Open Sci. (2021) 29:68–76. doi: 10.1016/j.euros.2021.05.006 34337536 PMC8317829

[14] Martorana E Bruschi M Scialpi P Grisanti R Scialpi M. Oligometastatic prostate cancer: is there a role for surgery? A narrative review. Turk J Urol. (2022) 48:174–9. doi: 10.5152/tud.2022.22064 PMC9730266 35634935

[15] Parker CC James ND Brawley CD Clarke NW Hoyle AP Ali A. Radiotherapy to the primary tumour for newly diagnosed, metastatic prostate cancer (STAMPEDE): a randomised controlled phase 3 trial. Lancet. (2018) 392:2353–66. doi: 10.1016/S0140-6736(18)32486-3 PMC6269599 30355464

[16] Boevé LMS Hulshof M Vis AN Zwinderman AH Twisk JWR Witjes WPJ. Effect on survival of androgen deprivation therapy alone compared to androgen deprivation therapy combined with concurrent radiation therapy to the prostate in patients with primary bone metastatic prostate cancer in a prospective randomised clinical trial: data from the HORRAD trial. Eur Urol. (2019) 75:410–8. doi: 10.1016/j.eururo.2018.09.008 30266309

[17] Burdett S Boevé LM Ingleby FC Fisher DJ Rydzewska LH Vale CL. Prostate radiotherapy for metastatic hormone-sensitive prostate cancer: A STOPCAP systematic review and meta-analysis. Eur Urol. (2019) 76:115–24. doi: 10.1016/j.eururo.2019.02.003 PMC6575150 30826218

[18] Madhavan K Jena R Marathi VR Kaushal D Deen S Rustagi S. Does radical local treatment in oligometastatic prostate cancer improve overall survival: A systematic review and meta-analysis. Urology. (2023) 182:5–13. doi: 10.1016/j.urology.2023.09.014 37774847

[19] Gratzke C Engel J Stief CG. Role of radical prostatectomy in clinically non-organ-confined prostate cancer. Curr Urol Rep. (2014) 15:455. doi: 10.1007/s11934-014-0455-9 25234189

[20] Jang WS Kim MS Jeong WS Chang KD Cho KS Ham WS. Does robot-assisted radical prostatectomy benefit patients with prostate cancer and bone oligometastases? BJU Int. (2018) 121:225–31. doi: 10.1111/bju.13992 28834084

[21] Heidenreich A Fossati N Pfister D Suardi N Montorsi F Shariat S. Cytoreductive radical prostatectomy in men with prostate cancer and skeletal metastases. Eur Urol Oncol. (2018) 1:46–53. doi: 10.1016/j.euo.2018.03.002 31100228

[22] Scharl S Hadaschik B Wiegel T Thomas C. Treatment of primary oligometastatic prostate cancer. Urologe A. (2021) 60:1527–33. doi: 10.1007/s00120-021-01643-0 34825936

[23] Connor MJ Smith A Miah S Shah TT Winkler M Khoo V. Targeting oligometastasis with stereotactic ablative radiation therapy or surgery in metastatic hormone-sensitive prostate cancer: A systematic review of prospective clinical trials. Eur Urol Oncol. (2020) 3:582–93. doi: 10.1016/j.euo.2020.07.004 32891600

[24] Miszczyk M Rajwa P Yanagisawa T Nowicka Z Shim SR Laukhtina E. The efficacy and safety of metastasis-directed therapy in patients with prostate cancer: A systematic review and meta-analysis of prospective studies. Eur Urol. (2024) 85:125–38. doi: 10.1016/j.eururo.2023.10.012 37945451

[25] Andrews JR Ahmed ME Sharma V Britton C Stish B Phillips R. Metastasis-directed therapy without androgen deprivation therapy in solitary oligorecurrent prostate cancer. J Urol. (2022) 208:1240–9. doi: 10.1097/JU.0000000000002898 36349914

[26] Ost P Reynders D Decaestecker K Fonteyne V Lumen N De Bruycker A. Surveillance or metastasis-directed therapy for oligometastatic prostate cancer recurrence: A prospective, randomized, multicenter phase II trial. J Clin Oncol. (2018) 36:446–53. doi: 10.1200/JCO.2017.75.4853 29240541

[27] Steuber T Jilg C Tennstedt P De Bruycker A Tilki D Decaestecker K. Standard of care versus metastases-directed therapy for PET-detected nodal oligorecurrent prostate cancer following multimodality treatment: A multi-institutional case-control study. Eur Urol Focus. (2019) 5:1007–13. doi: 10.1016/j.euf.2018.02.015 29530632

[28] Phillips R Shi WY Deek M Radwan N Lim SJ Antonarakis ES. Outcomes of observation vs stereotactic ablative radiation for oligometastatic prostate cancer: the ORIOLE phase 2 randomized clinical trial. JAMA Oncol. (2020) 6:650–9. doi: 10.1001/jamaoncol.2020.0147 PMC7225913 32215577

[29] Pellegrino A Gandaglia G de Angelis M Fallara G Mazzone E Stabile A. Oncological and perioperative outcomes of surgery with or without metastasis-directed therapy as part of a multimodal treatment in men with *de-novo* oligometastatic prostate cancer. World J Urol. (2023) 41:2069–76. doi: 10.1007/s00345-023-04460-6 37326656

[30] Schick U Jorcano S Nouet P Rouzaud M Vees H Zilli T. Androgen deprivation and high-dose radiotherapy for oligometastatic prostate cancer patients with less than five regional and/or distant metastases. Acta Oncol. (2013) 52:1622–8. doi: 10.3109/0284186X.2013.764010 23544357

[31] Gandaglia G Fossati N Stabile A Bandini M Rigatti P Montorsi F. Radical prostatectomy in men with oligometastatic prostate cancer: results of a single-institution series with long-term follow-up. Eur Urol. (2017) 72:289–92. doi: 10.1016/j.eururo.2016.08.040 27574820

[32] Mandel P Steuber T Graefen M. Radical prostatectomy in oligometastatic prostate cancer. Curr Opin Urol. (2017) 27:572–9. doi: 10.1097/MOU.0000000000000445 28825924

[33] Sooriakumaran P Wilson C Rombach I Hassanali N Aning J A DL. Feasibility and safety of radical prostatectomy for oligo-metastatic prostate cancer: the Testing Radical prostatectomy in men with prostate cancer and oligo-Metastases to the bone (TRoMbone) trial. BJU Int. (2022) 130:43–53. doi: 10.1111/bju.v130.1 34878715

[34] Sooriakumaran P Karnes J Stief C Copsey B Montorsi F Hammerer P. A multi-institutional analysis of perioperative outcomes in 106 men who underwent radical prostatectomy for distant metastatic prostate cancer at presentation. Eur Urol. (2016) 69:788–94. doi: 10.1016/j.eururo.2015.05.023 26038098

[35] Knipper S Graefen M. Primary tumor treatment in oligometastatic prostate cancer: radiotherapy versus radical prostatectomy. Eur Urol Open Sci. (2022) 35:68–9. doi: 10.1016/j.euros.2021.06.015 PMC8738893 35024634

[36] Parker CC James ND Brawley CD Clarke NW Ali A Amos CL. Radiotherapy to the prostate for men with metastatic prostate cancer in the UK and Switzerland: Long-term results from the STAMPEDE randomised controlled trial. PloS Med. (2022) 19:e1003998. doi: 10.1371/journal.pmed.1003998 35671327 PMC9173627

[37] Kadono Y Nohara T Ueno S Izumi K Kitagawa Y Konaka H. Validation of TNM classification for metastatic prostatic cancer treated using primary androgen deprivation therapy. World J Urol. (2016) 34:261–7. doi: 10.1007/s00345-015-1607-3 26047654

[38] Chaloupka M Herlemann A Spek A Gratzke C Stief C. Cytoreductive, radical prostatectomy in metastatic prostate cancer. Urologe A. (2017) 56:1430–4. doi: 10.1007/s00120-017-0505-2 28983651

[39] Knipper S Beyer B Mandel P Tennstedt P Tilki D Steuber T. Outcome of patients with newly diagnosed prostate cancer with low metastatic burden treated with radical prostatectomy: a comparison to STAMPEDE arm H. World J Urol. (2020) 38:1459–64. doi: 10.1007/s00345-019-02950-0 31511970

[40] Satkunasivam R Kim AE Desai M Nguyen MM Quinn DI Ballas L. Radical prostatectomy or external beam radiation therapy vs no local therapy for survival benefit in metastatic prostate cancer: A SEER-medicare analysis. J Urol. (2015) 194:378–85. doi: 10.1016/j.juro.2015.02.084 PMC4834920 25711194

[41] Ham WS Park JS Jang WS Kim J. Radical prostatectomy versus radiotherapy as local therapy for primary tumors in patients with oligometastatic prostate cancer. Front Oncol. (2024) 14:1368926. doi: 10.3389/fonc.2024.1368926 38544836 PMC10965631

[42] Gotto GT Yunis LH Vora K Eastham JA Scardino PT Rabbani F. Impact of prior prostate radiation on complications after radical prostatectomy. J Urol. (2010) 184:136–42. doi: 10.1016/j.juro.2010.03.031 20478594

[43] Francolini G Porreca A Facchini G Santini D Bruni A Simoni N. PERSIAN trial (NCT05717660): an ongoing randomized trial testing androgen deprivation therapy, apalutamide and stereotactic body radiotherapy. An alternative "triplet" for oligometastatic hormone sensitive prostate cancer patients. Med Oncol. (2023) 41:39. doi: 10.1007/s12032-023-02268-3 38157111

[44] O'Shaughnessy MJ McBride SM Vargas HA Touijer KA Morris MJ Danila DC. A pilot study of a multimodal treatment paradigm to accelerate drug evaluations in early-stage metastatic prostate cancer. Urology. (2017) 102:164–72. doi: 10.1016/j.urology.2016.10.044 PMC5468169 27888148

[45] Nientiedt C Budczies J Endris V Kirchner M Schwab C Jurcic C. Mutations in TP53 or DNA damage repair genes define poor prognostic subgroups in primary prostate cancer. Urol Oncol. (2022) 40:8.e11–8. doi: 10.1016/j.urolonc.2021.06.024 34325986

[46] Onal C Guler OC Erpolat P Hurmuz P Sutera P Deek MP. Evaluation of treatment outcomes of prostate cancer patients with lymph node metastasis treated with definitive radiotherapy: comparative analysis of PSMA PET/CT and conventional imaging. Clin Nucl Med. (2024) 49:e383–9. doi: 10.1097/RLU.0000000000005284 38847441

[47] de Bono J Mateo J Fizazi K Saad F Shore N Sandhu S. Olaparib for metastatic castration-resistant prostate cancer. N Engl J Med. (2020) 382:2091–102. doi: 10.1056/NEJMoa1911440 32343890

