# Supplementary material for: Improved survival of patients with newly diagnosed oligometastatic prostate cancer through intensified multimodal treatment
Source: Front Oncol. 2024 Dec 10;14:1475914. doi: 10.3389/fonc.2024.1475914 (PMC11666478; doi:10.3389/fonc.2024.1475914)
Supplement: Supplementary file 1 [file DataSheet1.pdf]

Supplementary Table 1: Uni- and multivariate Cox regression analysis of progression free and cancer specific survival (PFS, CSS).

	Cox regression analysis (PFS)				Cox regression analysis (CSS)			
	Univariate		Multivariate		Univariate		Multivariate	
	HR (95% CI)	p-value	HR (95% CI)	p-value	HR (95% CI)	p-value	HR (95% CI)	p-value
Age (<70 vs. 70 years	0.268 (0.061-1.180)	0.082	0.168 (0.026 – 1.077)	0.060	-*	-	-*	-
PSA (<20 vs. ≥20 ng/ml)	1.184 (0.539 – 2.600)	0.674	0.878 (0.357 – 2.159)	0.777	2.218 (0.587 – 8.373)	0.240	2.030 (0.456 – 9.026)	0.353
BMI (<25 vs. ≥25)	1.275 (0.528 – 3.075)	0.589	1.513 (0.536 – 4.266)	0.434	0.982 (0.254 – 3.804)	0.979	0.681 (0.150 – 3.095)	0.619
Biopsy Grade Group (<4 vs. ≥4)	9.556 (1.286-71.012)	0.027	24.640 (2.370 - 256.149)	0.007	-*	-	-*	-
No. of bone metastases (<3 vs. ≥3)	0.912 (0.341 - 2.439)	0.854	0.242 (0.073 – 0.805)	0.021	1.126 (0.316 – 4.755)	0.769	0.838 (0.151 – 4.659)	0.840
pT Stage (≤3a vs. >3a)	2.188 (0.744 – 6.435)	0.155	1.559 (0.403 – 6.30)	0.520	1.321 (0.347 – 5.030)	0.684	0.571 (0.104 -3.123)	0.518
No. of positive lymph nodes (<2 vs. ≥2)	1.632 (0.699 – 3.808)	0.257	1.013 (0.340 – 3.016)	0.982	3.457 (0.729 – 16.402)	0.118	1.793 (0.296 -10.860)	0.525
RP Grade Group (<4 vs. ≥4)	2.49 (0.813 – 5.163)	0.128	0.515 (0.157 – 1.685)	0.273	4.887 (0.619 – 38.594)	0.132	2.358 (0.181 – 30.751)	0.513
No. of treatments (<4 vs. ≥4)	1.126 (0.503 – 2.518)	0.773	0.435 (0.156 – 1.215)	0.112	0.755 (0.195 – 2.927)	0.684	0.627 (0.122 – 3.221)	0.575

* Not applicable as no events occurred in one of the groups
